# Supplementary material for: The Systems Biology Research Tool: evolvable open-source software
Source: BMC Syst Biol. 2008 Jun 29;2:55. doi: 10.1186/1752-0509-2-55 (PMC2446383; doi:10.1186/1752-0509-2-55)
Supplement: Additional file 1 — SBRT Archive. An archive of the current version of the Systems Biology Research Tool. [file 1752-0509-2-55-S1.zip › sbrt-1.4.0/doc/developers_guide/api/sbrt/kernel/fba/proc/FluxCapProcess.html]

FluxCapProcess


|  |  |  |
| --- | --- | --- |
| **Overview** \| **Package** \| **Class** \| **Use** \| **Tree** \| **Deprecated** \| **Index** \| **Help** | |  |
| **PREV CLASS**   **NEXT CLASS** | **FRAMES**    **NO FRAMES**     **All Classes** |  |
| SUMMARY: NESTED \| FIELD \| CONSTR \| METHOD | DETAIL: FIELD \| CONSTR \| METHOD |  |


---


## sbrt.kernel.fba.proc Class FluxCapProcess

```
java.lang.Object
  sbrt.kernel.fba.proc.FbaProcess<java.util.Map<java.lang.String,java.util.Set<LinearComb<java.lang.String>>>>
      sbrt.kernel.fba.proc.FluxCapProcess
```

**All Implemented Interfaces:**: KernelProcess<java.util.Map<java.lang.String,java.util.Set<LinearComb<java.lang.String>>>>, MonitoredProcess<java.util.Map<java.lang.String,java.util.Set<LinearComb<java.lang.String>>>>

---

``` public final class FluxCapProcess extends FbaProcess<java.util.Map<java.lang.String,java.util.Set<LinearComb<java.lang.String>>>> implements MonitoredProcess<java.util.Map<java.lang.String,java.util.Set<LinearComb<java.lang.String>>>> ```

This class is used to perform a flux cap identification. It simply adds the
results of a FluxCapComputer to a ResultsManager.

**Author:**
:   This class was written and documented by Jeremiah Wright
    while in the Wagner lab.

---

**Constructor Summary**

`FluxCapProcess(FluxCapComputer computer)`
  
          Constructs a new flux cap process.


| **Method Summary** | |
| --- | --- |
| `Fluxome` | `getFluxome()`             Returns the fluxome for which flux caps are computed. |
| `ProgressMonitor` | `getProgress()`             Returns a progress monitor for this experiment. |
| `void` | `run(ResultsManager<java.util.Map<java.lang.String,java.util.Set<LinearComb<java.lang.String>>>> resultsManager)`             Computes the flux caps and adds the result to the provided manager. |

**Methods inherited from class sbrt.kernel.fba.proc.FbaProcess**

`toString`

**Methods inherited from class java.lang.Object**

`clone, equals, finalize, getClass, hashCode, notify, notifyAll, wait, wait, wait`

**Constructor Detail**

### FluxCapProcess

```
public FluxCapProcess(FluxCapComputer computer)
```

:   Constructs a new flux cap process.

    **Parameters:**: `computer` - the computer with which all flux caps will be computed.


**Method Detail**

### getFluxome

```
public Fluxome getFluxome()
```

:   Returns the fluxome for which flux caps are computed.

    :   **Specified by:**: `getFluxome` in class `FbaProcess<java.util.Map<java.lang.String,java.util.Set<LinearComb<java.lang.String>>>>`
    :   **Returns:**: the fluxome for which flux caps are computed.

---


### run

```
public void run(ResultsManager<java.util.Map<java.lang.String,java.util.Set<LinearComb<java.lang.String>>>> resultsManager)
```

:   Computes the flux caps and adds the result to the provided manager.

    :   **Specified by:**: `run` in interface `KernelProcess<java.util.Map<java.lang.String,java.util.Set<LinearComb<java.lang.String>>>>`
    :   **Parameters:**: `resultsManager` - the results manager to which the flux cap-results will be added.

---


### getProgress

```
public ProgressMonitor getProgress()
```

:   Returns a progress monitor for this experiment.

    :   **Specified by:**: `getProgress` in interface `MonitoredProcess<java.util.Map<java.lang.String,java.util.Set<LinearComb<java.lang.String>>>>`
    :   **Returns:**: a progress monitor for this experiment.


---


|  |  |  |
| --- | --- | --- |
| **Overview** \| **Package** \| **Class** \| **Use** \| **Tree** \| **Deprecated** \| **Index** \| **Help** | |  |
| **PREV CLASS**   **NEXT CLASS** | **FRAMES**    **NO FRAMES**     **All Classes** |  |
| SUMMARY: NESTED \| FIELD \| CONSTR \| METHOD | DETAIL: FIELD \| CONSTR \| METHOD |  |


---
